# Continuous production of indole-3-acetic acid by immobilized cells of *Arthrobacter agilis*

**DOI:** 10.1007/s13205-017-0605-0

**Published:** 2017-04-11

**Authors:** Murat Ozdal, Ozlem Gur Ozdal, Alev Sezen, Omer Faruk Algur, Esabi Basaran Kurbanoglu

**Affiliations:** Department of Biology, Faculty of Science, Ataturk University, 25240 Erzurum, Turkey

**Keywords:** Indole acetic acid, Immobilized cells, Fermentation, *Arthrobacter agilis*

## Abstract

Indole acetic acid (IAA) is a plant growth-promoting hormone used in agriculture; therefore, its continuous production is of paramount importance. IAA-producing eight bacteria were isolated from the rhizosphere of *Verbascum vulcanicum.* Among them, *Arthrobacter agilis* A17 gave maximum IAA production (75 mg/L) and this strain was used to immobilization studies. The *A. agilis* A17 cells were immobilized in calcium alginate for the production of IAA. Optimization of process parameters for IAA production was carried out to enhance IAA production using immobilized cells. The maximal production of IAA was 520 mg/L under the following optimal conditions: 1% mannitol, 30 °C, pH 8.0, and 24 h incubation. It was determined that the immobilized cells could be reused (13 times) for the production of IAA.

## Introduction

Indole acetic acid (IAA) is a natural auxin which is produced by plants, bacteria and fungi. In plants, IAA is critical for plant growth and development. Most of the bacteria isolated from the rhizosphere can produce IAA (Duca et al. 2014; Patten and Glick 1996). Many researchers reported that plant growth-promoting bacteria from different genera (*Azospirillum*, *Bacillus*, *Enterobacter*, *Azotobacter*, *Klebsiella*, *Pseudomonas*, and *Arthrobacter*), Actinomycetes (*Streptomyces olivaceoviridis, S. rimosus*) and fungi (*Colletotrichum gloeosporioides, Ustilago maydis*) enhance plant growth by the synthesis of IAA (Khamna et al. 2010; Reineke et al. 2008).

IAA can be synthesized via either tryptophan-dependent pathway or tryptophan-independent pathway. The indole-3-acetamide, indole-3-pyruvate, tryptamine, tryptophan side-chain oxidase and indole-3-acetonitrile pathways are considered as main IAA biosynthesis through a tryptophan-dependent pathway in bacteria (Spaepen et al. 2007). Forni et al. (1992) and Yadav et al. (2015) reported that cells of *Arthrobacter* species (*A. sulfonivorans, A. sulfureus*, *A. globiformis, A. nicotianae, A. crystallopoietes*) produced IAA in the culture medium when precursor l-tryptophan was present in the medium. However, some bacteria have the tryptophan-independent pathway (starting from indole or indole-3-glycerol phosphate) to produce IAA (Arora and Bae 2014; Arora et al. 2015a).

Immobilized biocatalysts are widely used to produce different types of products and enzymes. They provide many benefits, such as higher stability, lower operational costs, continuous use of the biocatalysts, higher resistance to contamination, easier separation from the production medium and enhanced reaction yield (Kurbanoglu et al. 2010a, b; Okay et al. 2013). Different support materials (carrageenan, polyurethane, polyethylene glycol, and alginate) have been suggested for cell immobilization. Among them, sodium alginate is the most commonly used system because it is a rapid, nontoxic, low cost, and an easy method (Singh et al. 2012; Silbir et al. 2014; Yewale et al. 2016).

According to our knowledge, there is no earlier study using Ca-alginate immobilized *A. agilis* cells for IAA production. Considering the economic importance of IAA, this study aimed to continuous production of IAA with immobilized cells of *A. agilis* A17.

## Materials and methods

### Isolation of IAA producing bacteria from rhizospheric soil

Soil sample was collected from the rhizosphere of *Verbascum vulcanicum* from Palandöken Mountain, Turkey (39°49′15N, 41°17′34E). The isolation of microorganisms was done according to Mohite (2013). One gram of rhizospheric soil sample was suspended in 10 mL of sterile physiological water. It was incubated on rotary shaker at 150 rpm for 10 min. One mL of sample was serially diluted up to 10^−7^. One hundred microliter of diluted sample was plated on sterile Luria–Bertani (LB) agar medium containing 1 g/L l-tryptophan (Sigma) and incubated for 3 days at 30 °C. Single colonies were picked up and streaked on sterile LB agar plates to get pure culture. Total eight isolates were obtained from the rhizospheric soil and they were screened out for the production of IAA.

### Screening of isolates for indole acetic acid production

The isolates were grown in tryptic soy broth (TSB) on a rotary shaker at 150 rpm for 24 h at 30 °C. Bacterial isolates were evaluated for IAA production by inoculating 1 mL of each of cells suspension (1 × 10^6^ CFU/mL) in 50 mL of TSB (containing 1 g/L l-tryptophan) kept in 250-mL Erlenmeyer flasks. The flasks were incubated in the dark at 30 °C, 150 rpm, for 72 h.

### Determination of IAA production

After incubation periods, the culture media were centrifuged (4000 rpm for 10 min). One mL of supernatant was combined with 2 ml of Salkowski’s reagent (49 mL 35% perchloric acid and 1 mL 0.5 M FeCl_3_) and incubated for 30 min at room temperature. The production of IAA was determined by colorimetric measurement at 530 nm using Salkowski’s reagent as described by Patten and Glick (2002). The quantity of IAA was determined by comparison with a standard curve using pure IAA.

### Bacterial identification

The bacterial isolates were preliminarily characterized by Gram’s staining. The selected bacterium producing the highest IAA was then identified based on the 16S rDNA sequencing using universal primer set 27F (5′-AGAGTTTGATCMTGGCTCAG-3′) and 1102R (5′-GAGGTTCTGTGCTCCTCAGC-3′) (Gur et al. 2014). The isolate identification was verified by the analysis of 16S rDNA sequence (RefGen Life Sciences, Ankara, Turkey) which was compared with the National Center for Biotechnology Information (NCBI) database using the BLAST search on the web site (http://www.ncbi.nlm.nih.gov/BLAST) and submitted to GenBank. This bacterium was also identified according to its cytological and metabolic features, such as pigment formation, motility, spore formation, Gram staining, nitrate reduction, catalase and oxidase tests, and starch hydrolysis. These tests were carried out according to the Harley and Prescott (2002).

### Immobilization of *Arthrobacter agilis* A17 cells

*Arthrobacter agilis* cells were immobilized in calcium alginate gel beads as described previously (Kurbanoglu et al. 2010a; Okay et al. 2013). *Arthrobacter agilis* A17 was grown in Nutrient Broth (Merck) at 28 °C for 24 h. The culture was centrifuged at 5000 rpm for 15 min and the biomass was washed two times with sterile 0.85% (w/v) saline solution (SSS). Wet cells (4 g) were thoroughly resuspended in 40 mL of SSS and the total volume was completed to 50 ml with SSS. Sodium alginate solution (3%, w/v) was prepared by dissolving sodium alginate in SSS at 70 °C. The cell suspension was mixed with an equal volume of sodium alginate solution and stirred for 5 min. The mixture was dropped into a well stirred sterile CaCl_2_ solution (3.5%, w/v) using a syringe. Each alginate drop solidified upon contact with CaCl_2_ and formed beads that encapsulated the *Arthrobacter agilis* A17 cells. The beads were left to harden for 30 min at room temperature after they were washed with SSS to remove excess calcium ions and un-encapsulated cells. The average bead diameter was approximately 2–3 mm.

### Optimization of reaction parameters

In dark conditions, the immobilized cells (3.5 g) were incubated in 50 mM Tris buffer containing 1 g/L l-tryptophan on a rotary shaker at 150 rpm. Optimization of all variables was performed with 25 mL of reaction mixture in a 250-mL Erlenmeyer flask. To optimize temperature, the reaction was performed at pH 7.2 and 150 rpm with temperatures varying between 18 and 36 °C. For pH optimization, the conditions were 30 °C, 150 rpm, and Tris buffer pH 6–9. Different carbon sources (glucose, lactose, sucrose, maltose, mannitol, and fructose) were screened for IAA production using immobilized beads. Effect of incubation period (6–36 h) on IAA production was studied by immobilized cells.

### Statistical analysis

The statistical analyses of the data were performed using one-way analysis of variance (ANOVA). The level of significance was *p* < 0.05. Statistical analyses were conducted using the SPSS 20.0 software program.

## Results and discussion

### Isolation and screening of the bacteria for IAA production

**Fig. 1:**
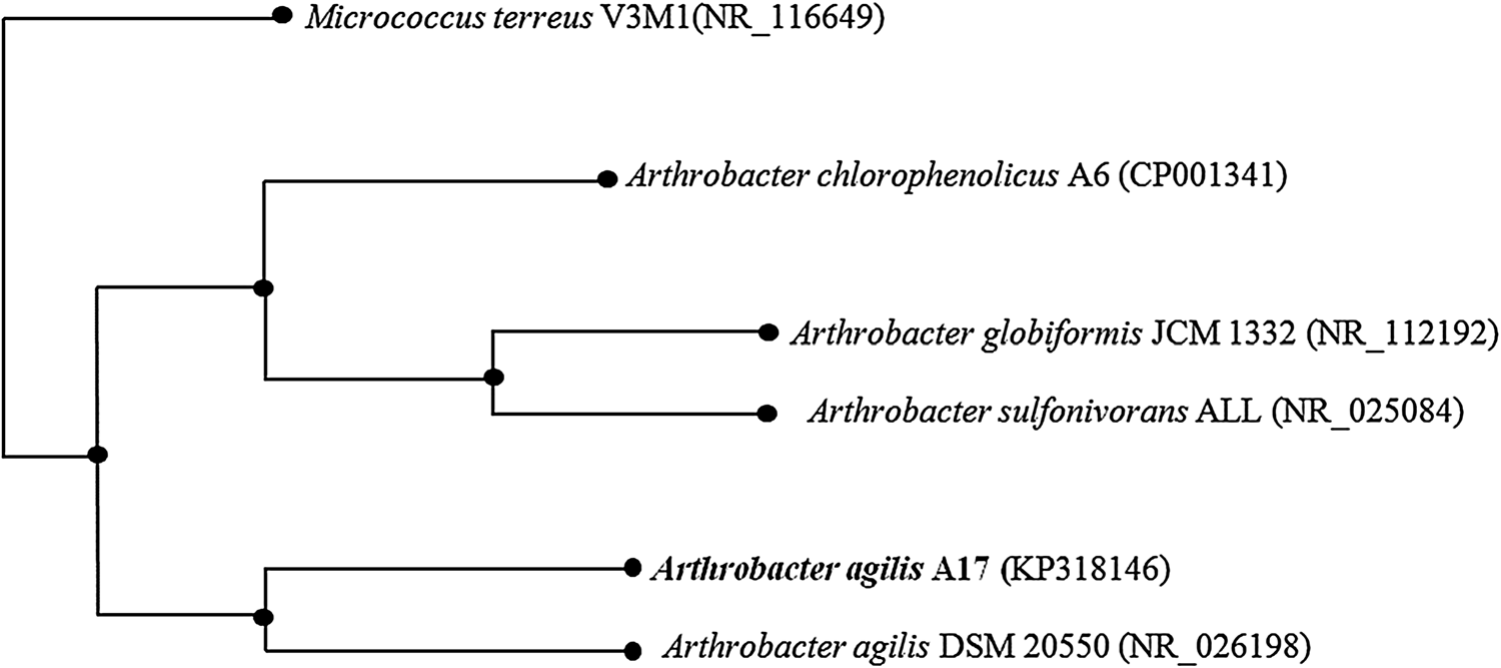
Phylogenetic tree based on the 16s rDNA sequences of strain A17 and related species

There are many different microorganisms in nature. IAA producers from the rhizosphere are more efficient than the others. It has been reported that 80% of bacteria isolated from the rhizosphere can produce the IAA (Patten and Glick 1996). In this study, a total of eight bacteria were isolated from the rhizospheric soil of *Verbascum vulcanicum*, comprising five isolates of Gram-negative and three Gram-positive bacteria. The range of IAA production in TSB with 1 g/L l-tryptophan was 14–75 mg/L. From the eight isolates obtained during screening, isolate A17 gave the best result for IAA production. Therefore, it was selected for subsequent studies. The data of the results from other isolates were not shown. *A. agilis* A17 was identified based on the 16S rDNA sequencing. Finally, 1442 bp 16S rDNA sequence of the strain was BLAST searched and aligned with *Arthrobacter* sequences. The sequence was deposited in GenBank with the accession number of KP318146. Nucleotide sequences were compared with NCBI GenBank entries and the similarities were determined by the BLAST algorithm (Fig. 1). Morphological and biochemical tests showed that A17 was a Gram-positive, red pigmented, aerobic, catalase and oxidase positive, coccoid shaped, and mobile organism (Table 1).

**Table 1:** Results of morphological and biochemical tests for the isolate A17

Test	Reaction
Catalase	+
Colony color	Red
Form	Coccoid
Growth at 5% NaCl	−
Oxidase	+
Nitrate reduction	−
Urease	−
Motile	+
Spore formation	−
Gram reaction	+
Aerobic	+
Methyl red	−
Gelatin hydrolysis	+
Starch hydrolysis	+
Esculin hydrolysis	+

+, positive result; −, negative result

*Arthrobacter* species (pigmented Gram-positive bacteria) have been isolated from different sources, such as soil, water, foods, radioactive waste and arctic ice. It appears that beta carotene offers protection for *A. agilis* in the natural environment (Dieser et al. 2010; Sutthiwong et al. 2014). Many species of *Arthrobacter* have been reported to produce IAA in the presence of l-tryptophan. *Arthrobacter globiformis* and *A. nicotianae* produced 10.1 and 4.4 mg/L IAA (Forni et al. 1992). Yuan et al. (2011) reported that *Arthrobacter* sp. produced 87.7 mg/L IAA. Similarly, Yadav et al. (2015) showed that *A. sulfonivorans*, *A. sulfureus* and *Arthrobacter* sp. produced 27.6, 48.2 and 28.2 mg/L IAA, respectively.

### Optimization of IAA production under immobilization conditions

Reaction conditions are significant for the successful production of IAA and optimization of parameters, such as temperature, pH, media composition and process time are essential in developing the process.

### Effect of temperature on IAA production

**Fig. 2:**
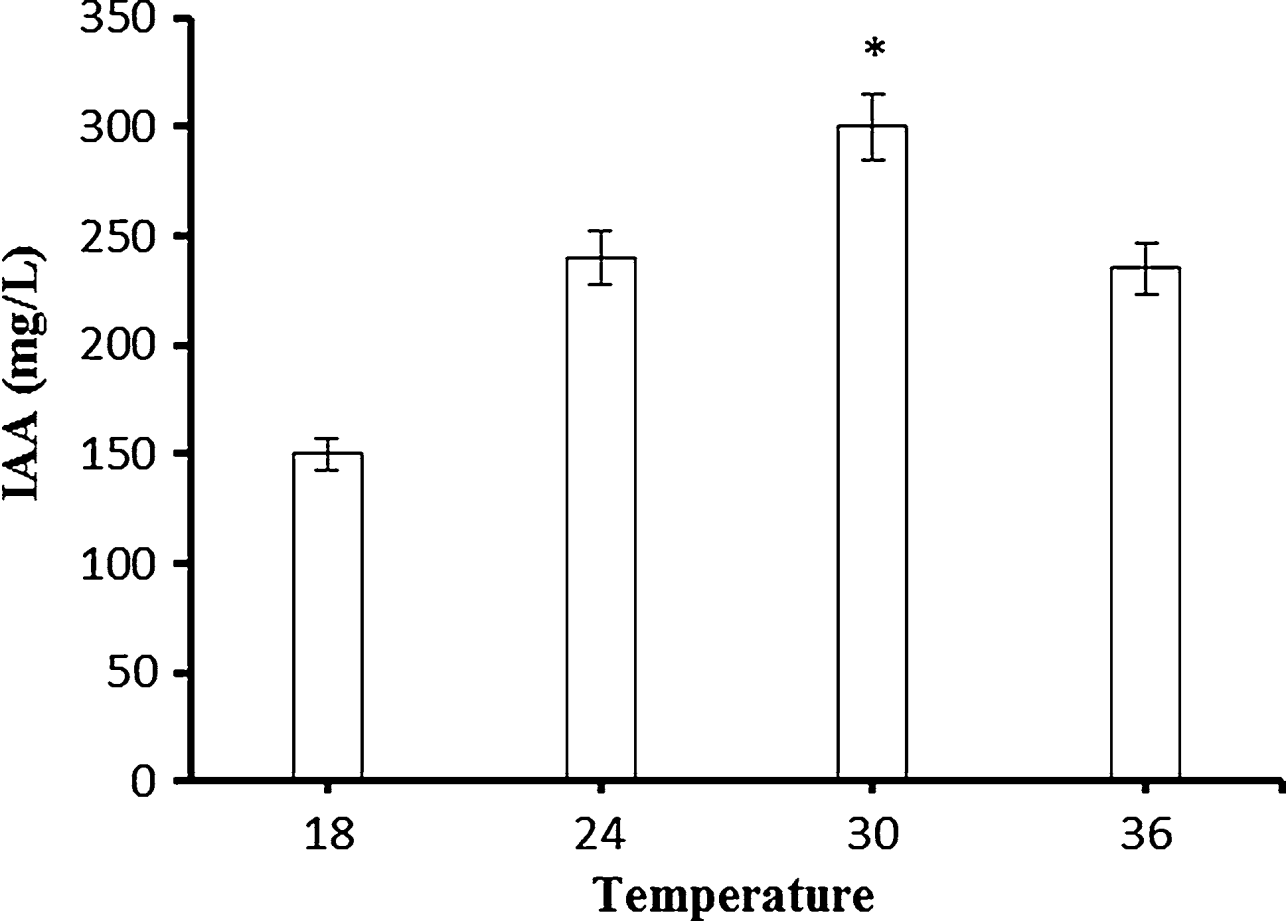
Effect of temperature on IAA production by Ca-alginate immobilized *A. agilis* A17. Reaction conditions: *T* pH 7.2, 150 rpm, 24 h,  %1 Glucose. An *asterisk* (*) denotes a value significantly greater than the other values (*p* < 0.05)

To find out the optimal temperature for IAA production, immobilized cells were cultivated at various temperatures ranging from 18 to 36 °C. The maximum IAA production (300 mg/L) was obtained when the immobilized cells were incubated at 30 °C. Temperatures above or below 30 °C did not enhance the IAA production (Fig. 2). Similar results were obtained with bacterial strains, namely *Pantoea agglomerans* PVM (Apine and Jadhav 2011), *Streptomyces viridis* CMU-H009 (Khamna et al. 2010), *Bacillus megaterium* and *Lactobacillus casei* (Mohite 2013). However, *Bacillus licheniformis* produced higher amounts of IAA at 37 °C (Prashanth and Mathivanan 2010).

### Effect of buffer pH on IAA production

**Fig. 3:**
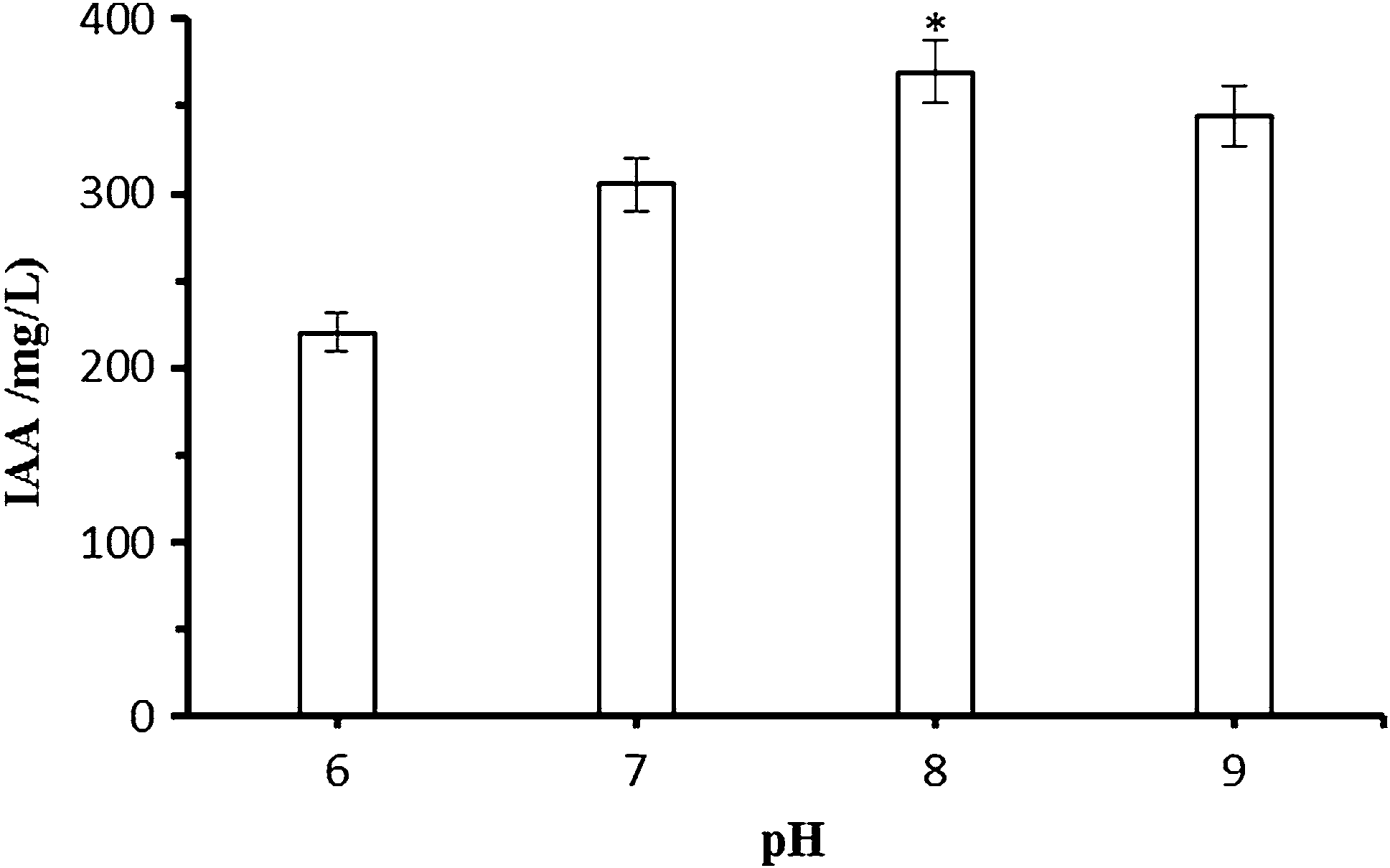
Effect of initial pH on IAA production by Ca-alginate immobilized *A. agilis* A17. Reaction conditions: *T* 30 °C, 150 rpm, 24 h,  %1 Glucose. An *asterisk* (*) denotes a value significantly greater than the other values (*p* < 0.05)

To investigate the effect of initial pH on IAA production, immobilized cells were cultivated with different initial buffer pHs (6.0–9.0). In this study, maximum IAA concentration (370 mg/L) was obtained at an initial pH 8.0, but no statistically significant difference between pH 8.0 and pH 9.0 (Fig. 3). A lower production of IAA (220 mg/L) was found when the immobilized cells were incubated at pH 6. Mohite (2013) reported that the synthesis of the highest IAA level was determined in cultures (*Bacillus megaterium, B. subtilis, Lactobacillus casei*) cultivated in an alkaline media at a pH 8. Maximal IAA production in *Pantoea agglomerans* PVM was at pH 7 (Apine and Jadhav 2011) and at pH 8 in *Klebsiella pneumoniae* K8 (Sachdev et al. 2009). It was found that the best IAA formation by *Streptomyces viridis* CMU-H009 was obtained at a pH 7 (Khamna et al. 2010). The pH and temperature can affect the activity of enzymes involved in the biosynthesis of IAA (Duca et al. 2014).

### Effect of carbon sources on IAA production

**Fig. 4:**
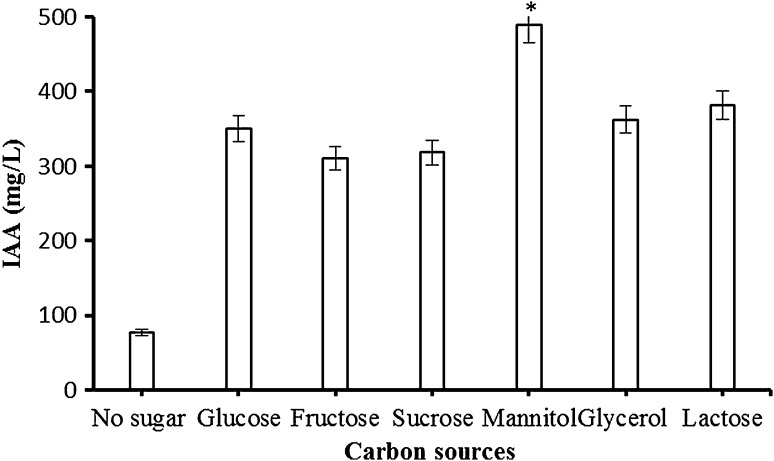
Effect of carbon sources on IAA production by Ca-alginate immobilized *A. agilis* A17. Reaction conditions: *T* 30 °C, pH 8, 150 rpm, 24 h. An *asterisk* (*) denotes a value significantly greater than the other value (*p* < 0.05)

The effect of carbon sources on the production of IAA by immobilized cells is shown in Fig. 4. When a variety of carbon sources (1%) were added to the tris buffer, IAA production were stimulated. However, in the absence of carbon sources, little IAA (77 mg/L) production was observed. Carbon sources had a supporting effect on IAA synthesis due to providing energy for cells and improved cofactor recycling in the cells (Singh et al. 2012). As shown in Fig. 4, a high level of IAA (490 mg/L) was obtained when mannitol was added as the carbon source. Many researchers reported that carbon sources addition in culture medium increased IAA synthesis (Datta and Basu 2000; Nutaratat et al. 2015).

### Effect of fermentation time on IAA production

**Fig. 5:**
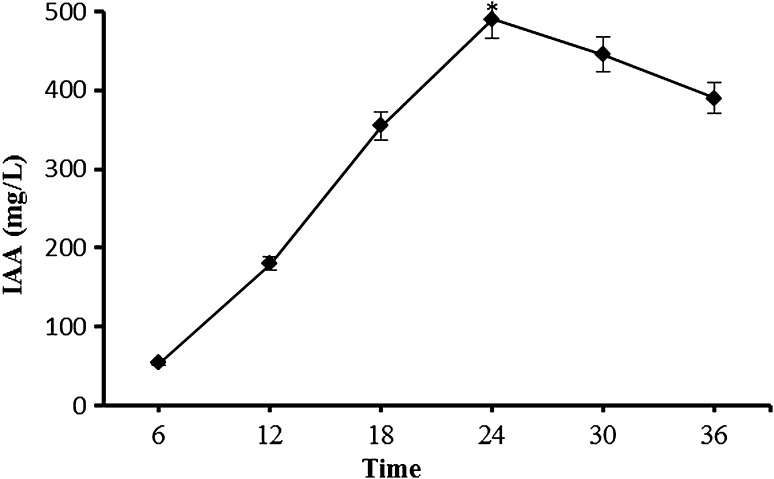
Effect of reaction time on the production of IAA by Ca-alginate immobilized *A. agilis* A17. Reaction conditions: *T* 30 °C, pH 8, 150 rpm, %1 mannitol. An *asterisk* (*) denotes a value significantly greater than the other values (*p* < 0.05)

The results of IAA production by immobilized *A. agilis* A17 cells at varying time periods is shown in Fig. 5. There was a gradual increase in IAA production from 6 to 24 h. The highest concentration of IAA (490 mg/L) was obtained at 24 h of fermentation but significantly decreased beyond this time. The decrease in IAA production after 24 h might be due to the synthesis of IAA degrading enzymes such as IAA oxidase and peroxidase (Arora et al. 2015b; Datta and Basu 2000). Leveau and Lindow (2005) reported degradation of IAA to catechol by *Pseudomonas putida*. Arora and Bae (2014) showed that *Arthrobacter* sp. SPG converted IAA to indole-3-glyoxylic acid and then to indole-3-aldehyde. Arora et al. (2015a) reported that *Lysinibacillus xylanilyticus* degraded IAA into 3-methylindole.

### Reusability of immobilized *Arthrobacter agilis* cells

**Fig. 6:**
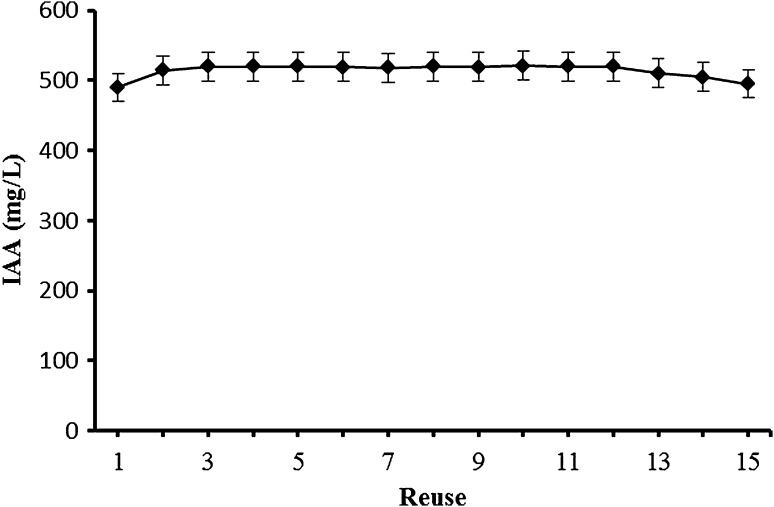
Reusability of the immobilized cells of *A. agilis* A17. *A. agilis* A17 cells were immobilized in 3.5% alginate gel and incubated in 100 mM Tris buffer containing %1 mannitol at 30 °C for 15  days, the buffer being changed daily

The immobilized cells were repeatedly used for the IAA production in the optimal conditions. Immobilized cells in alginate beads were reused in thirteen successive reaction cycles (each cycle 24 h) without any loss of biocatalytic activity (Fig. 6). The amount of IAA was found to be 490–520 mg/L during this period. In comparison with the conventional fermentations, the immobilization of microorganisms has many important advantages, such as higher cell density, reduced reaction time, long term reuse and higher product yield. For these reasons, immobilized cells have been used for various other valuable chemicals (Okay et al. 2013; Razak and Viswanath 2015).

## Conclusions

Screening experiments were carried out to select the most active bacterial isolate for IAA production. In this work, we successfully produced IAA using immobilized cells of *A. agilis* A17. The highest IAA was 490 mg/L for 24 h, at 1% mannitol concentration, 30 °C and pH 8. Moreover, our results showed that immobilized cells offers repeated use of the biocatalysts in the production of IAA. In subsequent studies, large scale application for IAA production will be developed with cells entrapped. In addition, high IAA-producing species (belonging to the genus *Enterobacter*, *Pantoea*, *Klebsiella*) should be investigated for overproduction of IAA with immobilized cells. The results obtained in the study suggested that production of IAA by immobilized *A. agilis* A17 appeared to be feasible method to continuous production of IAA.
